# Polyelectrolyte Threading through a Nanopore

**DOI:** 10.3390/polym8030073

**Published:** 2016-03-03

**Authors:** Pai-Yi Hsiao

**Affiliations:** Department of Engineering and System Science, National Tsing Hua University, Hsinchu 30013, Taiwan; pyhsiao@ess.nthu.edu.tw; Tel.: +886-3-516-2247

**Keywords:** polyelectrolyte, translocation, scaling behavior, conformation, probability distribution, density distribution, ion condensation, molecular simulations

## Abstract

Threading charged polymers through a nanopore, driven by electric fields *E*, is investigated by means of Langevin dynamics simulations. The mean translocation time 〈τ〉 is shown to follow a scaling law $N^{\alpha }$, and the exponent *α* increases monotonically from 1.16(4) to 1.40(3) with *E*. The result is double-checked by the calculation of mean square displacement of translocation coordinate, which asserts a scaling behavior $t^{\beta }$ (for *t* near *τ*) with *β* complying with the relation αβ=2. At a fixed chain length *N*, 〈τ〉 displayed a reciprocal scaling behavior $E^{-1}$ in the weak and also in the strong fields, connected by a transition $E^{-1.64(5)}$ in the intermediate fields. The variations of the radius of gyration of chain and the positions of chain end are monitored during a translocation process; far-from-equilibrium behaviors are observed when the driving field is strong. A strong field can strip off the condensed ions on the chain when it passes the pore. The total charges of condensed ions are hence decreased. The studies for the probability and density distributions reveal that the monomers in the trans-region are gathered near the wall and form a pancake-like density profile with a hump cloud over it in the strong fields, due to fast translocation.

## 1. Introduction

The study of the transport of polymers through nanometer-sized pores has attracted much attention in the scientific community and applications [1,2,3,4,5,6,7,8]. The story starts in 1994, with the first detection of polymers passing through pores by Bezrukov *et al.* [9]. Later, Kasianowicz *et al.* showed the promise of the idea to be a fast sequencing method for polynucleotide molecules [10]. It has triggered a great deal of experimental, theoretical, and simulation investigations until today, and renders nanopore research the “Holey Grail” in nanotechnology [11]. Despite of the efforts, scientists have not yet reached consensus on statistical properties and mechanisms of polymer translocation [3,6,7].

Polymer translocation was initially treated as a quasi-equilibrium process, and theoretically reduced to a drift-diffusion problem using the Fokker-Planck equation [12,13,14]. For unbiased translocation, the translocation time *τ* was shown to scale as $N^{2}/D$ where *N* is the chain length and *D* is the diffusion coefficient of chain. In the early work, Sung and Park [12] set $D∼N^{-1}$ for the case that the hydrodynamic interaction can be neglected (called “Rouse dynamics”) and $D∼N^{-1/2}$ for the one wherein hydrodynamics is considered (called “Zimm dynamics”), and obtained the scaling $\tau ∼N^{\alpha }$ with α=3 and α=2.5, respectively. Muthukumar [13] speculated that *D* should be the one of the monomers situated in the pore, and predicted α=2 since *D* is independent of *N*. Chuang *et al.* [15] later questioned about the quasi-equilibrium assumption and provided a lower bound for *α* to be 1+2ν. Dubbeldam *et al.* [16] used a fractional Fokker-Plank equation to include the non-equilibrium characteristics, and derived $\alpha =2+2\nu -\gamma_{1}≃2.49$ where $\gamma_{1}$ is the surface entropic exponent. Panja *et al.* [17,18,19] accounted for the memory effect arising from crowding and tension imbalance of chain across nanopores, and showed the scaling behavior with α=2+ν and 1+2ν for Rouse and Zimm dynamics, respectively. In parallel, the exponent *α* obtained by simulations are not fully in agreement with each other. The values fall mainly between 2 and 2.75 for the studies in 2D and 3D spaces (refer to the tables in the reviews [3,6,7]). Slater and coworkers [20,21,22] have pointed out that translocation depends heavily on the specifications of the system. For example, the translocation time *τ* decreases with increasing the pore size, but the exponent *α* increases with it. If the viscosity of the solvent is increased, both *τ* and *α* increase. Therefore, threading phenomena cannot be described simply in the framework of a universal behavior.

For biased translocation, experiments have reported various values of *α*: 1.27(3) [23,24, 1.34(15) [25], and 1.40(5) [26]. The threading speed of the chain is found to nonlinearly increase with the applied voltage [27], while the translocation time is inversely proportional to it [10,26,28]. If the temperature is increased, the translocation time decreases [26,29,30]. In theoretical studies, Sung and Park [12] predicted α=2 for Rouse chains and α=1.5 for Zimm chains at the first place. The analysis of Lubensky and Nelson [14] and Muthukumar [13] showed that $\tau ∼N^{\alpha }f^{-\delta }$ with α=1 and δ=1 when the driving force *f* is strong. As a result of non-equilibrium features, Kantor and Kardar [31] set a lower bound for the translocation, $\tau \geq N^{1+\nu }f^{-1}$. Vocks *et al.* [32] further provided a more accurate bound, on the basis of energy conservation, to be $\tau \geq \eta N^{2\nu }f^{-1}$ for Rouse dynamics and $\tau \geq \eta N^{3\nu -1}f^{-1}$ for Zimm, where *η* is the viscosity of solvent. By taking into account the memory decay, they derived α=(1+2ν)/(1+ν) and α=3ν/(1+ν) for the two dynamics. Sakaue and co-workers [33,34,35] predicted that the chain shape varies from a coiled conformation in a weakly-biased translocation to a trumpet, a stem-flower, and a strong-stretching conformation, in turn, as the driving force increases. Rowghanian and Grosberg [36] introduced the concept of iso-flux by keeping mass flow conserved, and predicted $\tau ∼N^{1+\nu }f^{-1}$ and $\tau ∼N^{1+\nu }f^{-(2\nu -1)/\nu }$ for Rouse and Zimm dynamics, respectively. Dubbeldam *et al.* [37] applied tensile blob analysis and claimed a transition of the scaling behavior from $\tau ∼N^{2\nu }f^{-1/\nu }$ to $\tau ∼N^{1+\nu }f^{-1}$ as *f* increases. When considering the cis–trans dynamical asymmetry (*i.e.*, the pulling of chain on the cis side versus the pushing of chain on the trans side), Saito and Sakaue [38] obtained the force exponent $\delta =p_{z}-p_{v,1}$ where $p_{z}$ and $p_{v,1}$ are two dynamical exponents. Many simulation efforts have also been devoted to searching for the exponents. Most of the reported values stay in the range: 1≤α≤1.75 and δ≃1. Readers can refer to the review papers [3,6,7] for a summary of recent results.

A simulation study of polymer translocation was performed in two-dimensional (2D) space at the beginning, for the sake of the limitation of computing resources and also for the verification of theories [15,31,39,40,41,42,43,44]. Today, 3D models have been widely used and most of them were done using coarse-grained models [22,32,45,46,47,48,49,50]. Langevin dynamics simulation is a frequently-employed method [22,41,47,49,50,51], in which the hydrodynamic interaction is ignored. To account for hydrodynamics, explicit solvent method [20,52], solvent stochastic rotation dynamics [53,54], dissipative particle dynamics [55,56,57], or lattice Boltzmann method [58,59,60,61,62] were performed. Some of the studies were able to investigate translocation behavior using all-atom models [52,63,64,65]. For unbiased translocation, part of a chain was threaded through a pore initially; the translocation occurs spontaneously owing to the entropic force which drives the chain out of the pore to decrease the free energy [39,40,45,66]. For biased translocation, a driving force was applied, usually inside the pore, to simulate electric-field driving [49,51,67]. Alternative models, such as driving chains in a solvent flow through a pore [48,57,68] or pulling chains directly on the head monomer [69,70] have been investigated. Since it is of little chance to pursue a universal behavior, researchers now pay more attention to the facts which influence translocation, such as solvent quality [22,55,56], pore-chain interaction [44,71,72,73], temperature [30,74,75], and geometrical effect of confined spaces [76,77,78,79]. Recently, experiments [80,81,82] and simulations [52,65,83,84,85] have shown a promising future for DNA sequencing through a graphene nanopore. Review papers focusing on experimental studies of DNA translocation and sensing using nanopores can be found at many places, for example, references [4,5,8,86,87,88,89].

In the above contributions, most simulations studied translocation behavior using neutral chains. If charged chains were used, the ions were usually not involved in the study and the electrostatic interaction between monomers were modeled by a mean-field (Debye–Huckel) potential $U_{\mathrm{DH}}\left(r\right)=q_{1}q_{2}\exp\left(-\kappa r\right)/\left(4\pi \epsilon r\right)$, where the screening effect of ions is effectively accounted through the exponential factor with $\kappa^{-1}$ being the Debye length [47]. There are only few studies, up to now, which explicitly involve the ions in the simulations [62,64]. It is known that in a polyelectrolyte solution, considerable counterions are condensed on the charged chains [90,91], which can dramatically change the conformation of the chain and affect the properties of the solution [92,93,94,95,96]. When a chain passes through a pore, the condensed-ion atmosphere can be disrupted by the pore geometry. Therefore, the ions should play an important role in the determination of translocation dynamics, and should not be neglected in the investigation. In order to properly take the influence of ions into account, we perform molecular simulations of single polyelectrolytes driven through a nanopore by electric fields, with the ions explicitly modeled. The organization of the paper is given as follows. The model and setup are described in Section 2. The translocation time and the distribution are studied in Section 3.1, to understand the dependence on chain length and field strength. The radii of gyration for chain segments in different space regions and the positions of the chain are calculated (Section 3.2). The translocation coordinate is investigated in Section 3.3, which double-checks the scaling behavior. The ion condensation is studied and the variation of the charge of the condensed ions is presented in Section 3.4. The probability distributions and density distributions are given in Section 3.5, which shows the detailed evolution of different components in the system during a translocation process. We give our conclusions in Section 4.

## 2. Model and Setup

**Figure 1:**
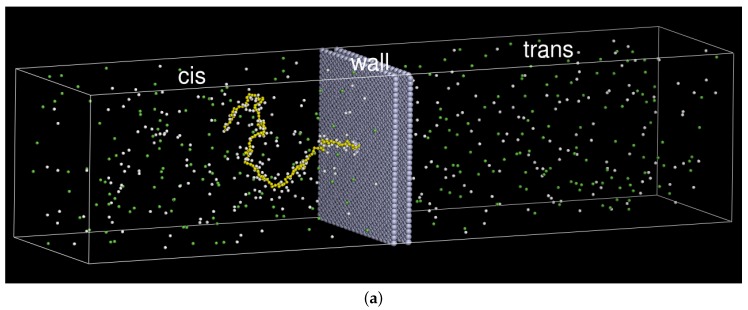

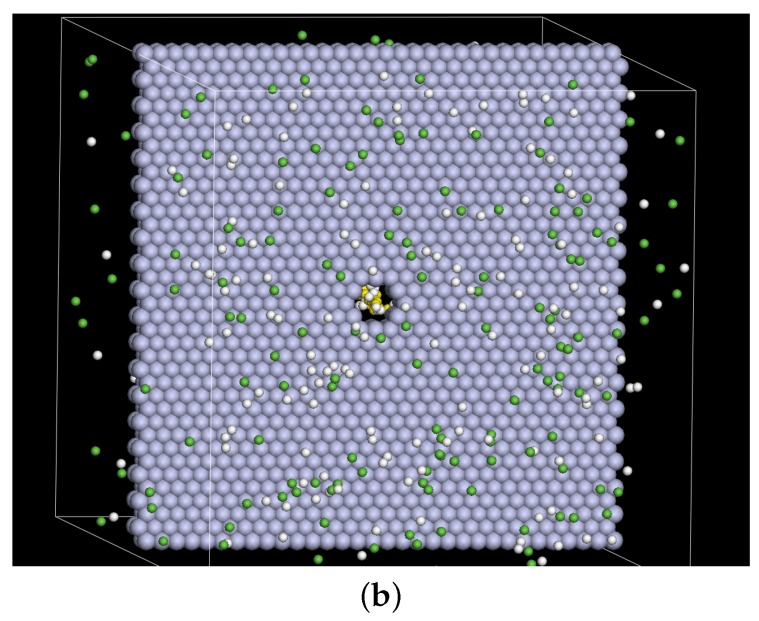
(**a**) Snapshot of a system of N=128, where *N* refers to the number of monomers; (**b**) the membrane wall viewed from the trans side of the system. A pore is punched through the wall at the center. The gray beads represent the wall. The yellow, white, and green beads represent the monomers, cations, and anions, respectively.

A bead-spring chain model was used to simulate polyelectrolytes threading through a pore in solutions. The system comprises a negatively charged chain, ions, and a membrane wall. The chain is composed of *N* monomers. Each monomer carries a charge −e, and dissociates a monovalent cation (or “counterion”) into the solution to maintain the electro-neutrality. 256 monovalent cations and anions were further added in the system to mimic a saline solution at a fixed concentration. The membrane wall was modeled by four layers of immobile beads placed at hexagonal lattice points. It divides the space into two compartment spaces: cis-region and trans-region, which are connected by a pore channel punched through the wall along the *z*-direction. To save computing resources, the interior beads of the wall were removed. The snapshots for a system of N=128 are given in Figure 1a,b.

The monomers and the ions were assumed to have the same mass *m* and size $\sigma_{\text{bb}}$. The excluded volume interaction between them was modeled by a short-range repulsive Lennard–Jones (LJ) potential

(1) $$U_{\text{bb}}\left(r\right)=\left\{\begin{array}{cc} 4\varepsilon_{\text{bb}}\left[{\left(\frac{\sigma_{\text{bb}}}{r}\right)}^{12}-{\left(\frac{\sigma_{\text{bb}}}{r}\right)}^{6}+\frac{1}{4}\right] & \text{if }r\leq \sqrt[{6}]{2}\sigma_{\text{bb}}\\ 0 & \text{if }r>\sqrt[{6}]{2}\sigma_{\text{bb}} \end{array}\right.$$

where *r* is the separation distance between two beads, $\sigma_{\text{bb}}$ represents the diameter of a bead, and $\varepsilon_{\text{bb}}$ is the interaction strength. We set $\sigma_{\text{bb}}=1.0\sigma$ and $\varepsilon_{\text{bb}}=1.2k_{B}T$, where *σ* is the length unit, *T* is the temperature, and $k_{B}$ is the Boltzmann constant. The excluded volume interaction between the monomer/ion beads and the wall beads, $U_{\text{bw}}\left(r\right)$, takes the same form as Equation (1), with $\sigma_{\text{bw}}=1.5\sigma$ and $\varepsilon_{\text{bw}}=2.5k_{B}T$. The bond connectivity between adjacent monomers on the chain was modeled by a harmonic potential

(2) $$U_{\text{bond}}\left(r\right)=\frac{1}{2}k{\left(r-r_{0}\right)}^{2}$$

where $k=600k_{B}T/\sigma^{2}$ is the spring constant and $r_{0}=1.0\sigma$ the equilibrium bond length. The charged beads interact with each other via the Coulomb interaction, expressed as

(3) $$U_{\text{coul}}\left(r\right)=k_{B}T\lambda_{B}\frac{Z_{i}Z_{j}}{r}$$

where $Z_{i}$ and $Z_{j}$ are the charge valences of the bead *i* and *j*, respectively, and $\lambda_{B}=e^{2}/\left(4\pi \epsilon k_{B}T\right)$, the Bjerrum length, defines the distance between two unit charges, at which the electrostatic energy is equal to the thermal energy $k_{B}T$. We set $\lambda_{B}=3.0\sigma$. Once $\lambda_{B}$ is set, the dielectric constant *ϵ* of solvent is set.

The whole system was placed in a rectangular box of dimension 48.0σ×49.36σ×200.0σ. Periodic boundary conditions were applied in the *x*, *y*, and *z*-directions. The Coulomb interactions were calculated using the particle–particle/particle–mesh Ewald method by setting the error tolerance equal to $10^{-3}$. The threading of the chain was driven by an electric field uniformly set inside the pore. The radius of the pore is 2.25σ and the length of the pore is 4.5σ. Langevin dynamics simulations were performed using the LAMMPS package [97]. The equation of motion reads as

(4) $$m\frac{d^{2}{\vec{r}}_{i}}{dt^{2}}=-\zeta \frac{d{\vec{r}}_{i}}{dt}-\nabla_{i}U+Z_{i}e\vec{E}\left({\vec{r}}_{i}\right)+{\vec{\eta }}_{i}$$

where *ζ* is the friction coefficient, *U* is the sum of the interaction potentials, $\vec{E}\left(\vec{r}\right)=-E\hat{z}$ is the applied electric field in the channel pore and points toward the $-\hat{z}$-direction, and ${\vec{\eta }}_{i}$ is a stochastic force. The first term on the right hand side of the equation accounts for the frictional force exerted on the bead when it moves through the solvent. The second term describes the conservative force acting on the bead. The stochastic term in the last takes into account the effect of random collisions given by solvent, because the solvent molecules were not modeled in the study. ${\vec{\eta }}_{i}$ has zero mean and satisfies the fluctuation-dissipation theorem $\langle {\vec{\eta }}_{i}\left(t\right)\cdot {\vec{\eta }}_{j}\left(t^{\prime }\right)\rangle =6k_{B}T\zeta \delta_{ij}\delta \left(t-t^{\prime }\right)$ [98], which served to control the system temperature. We set $\zeta =1.0\;m\tau_{u}^{-1}$ where $\tau_{u}=\sigma \sqrt{m/k_{B}T}$ is the time unit.

Initially, the first monomer of the chain was threaded through the pore and placed just at the entrance of the trans-region. The system was then equilibrated in zero fields under a constraint which keeps the first monomer immobile (refer to Figure 1). To start a translocation, we turned on the electric field and removed the constraint. The field acted on the monomers inside the pore, and the chain was driven through the pore to the trans-region. There exists a certain probability of a “failure of translocation”, in which a chain threads in a reverse direction instead of a forwarded one, back to the cis-region [47,56]. To guarantee a successful translocation, we imposed a LJ wall potential at the exit of the pore, visible only to the first monomer, which prevents the first monomer moving back into the pore. Physical quantities were calculated and collected during the translocation process. We varied the chain length from N=16 to 384 and the strength of the electric field from $E=0.2k_{B}T/e\sigma$ to $32.0k_{B}T/e\sigma$. For each case, at least 500 independent runs were performed. The equations of motion were integrated using the Verlet algorithm [99,100]. The time step of integration Δt was chosen between $0.0001\tau_{u}$ and $0.005\tau_{u}$, depending on the case.

To shorten the notation, the value of a physical quantity will be reported in (*σ*, *m*, $\tau_{u}$, *e*)-unit system in the following text, if the unit is not specified. For example, the concentration will be described in the unit $\sigma^{-3}$ and the energy in the unit $m\sigma^{2}\tau_{u}^{-2}\left(=k_{B}T\right)$. Since the Bjerrum length is 7.2Å in water and the room temperature T=300K is equivalent to an energy of $4.14\times 10^{-21}J$, our simulations can be mapped to a typical experimental condition by setting σ=2.4, $m=100g\cdot {\text{mol}}^{-1}$, and $\tau_{u}=1.5\text{ps}$. The parameters of simulation are summarized in Table 1.

## 3. Results and Discussion

### 3.1. Translocation Time

**Figure 2:**
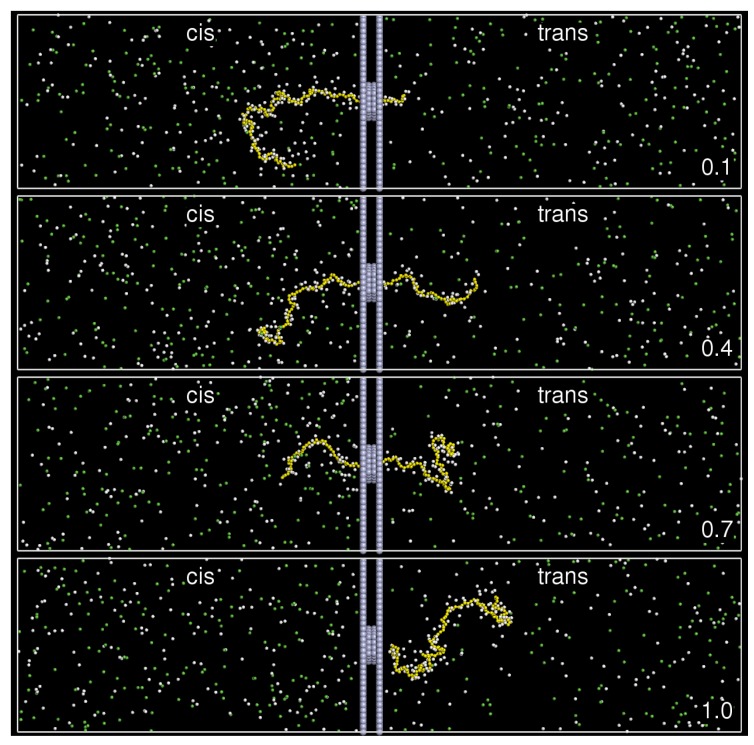
Snapshots of a typical translocation run for N=128 in E=0.5 at time t=0.1τ, 0.4τ, 0.7τ, and 1.0τ. The color scheme is the same as described in Figure 1.

Translocation time *τ* is defined as the time needed for a chain to complete a threading-through-the-pore process, starting from the switching-on of the electric field. The snapshots of a typical threading process are given in Figure 2, where a chain is seen to translocate from the left compartment (cis-region) to the right one (trans-region).

**Figure 3:**
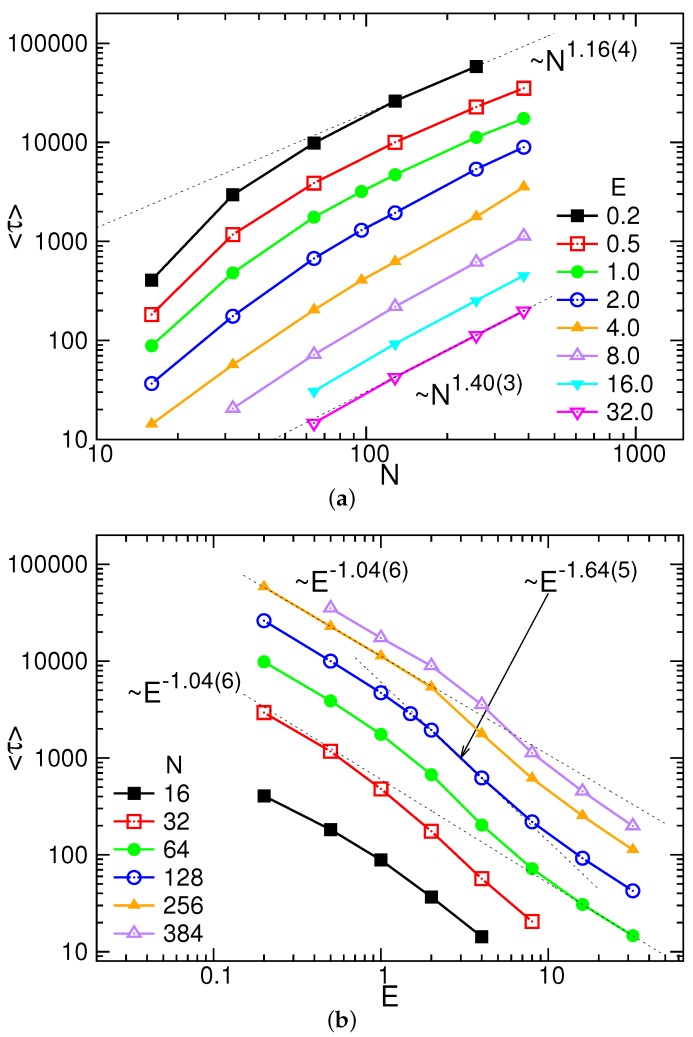
Mean translocation time 〈τ〉 as a function of (**a**) chain length *N* at a given *E*, and (**b**) field strength *E* at a given *N*. The error bar is smaller than the data symbol.

Since translocation is a diffusion-driven process, *τ* is the first passage time of the last monomer to leave the exit of the pore [101]. By performing many independent runs, we were able to calculate the mean translocation time 〈τ〉 for different *N* and *E*. The results are given in Figure 3.

As expected, the mean translocation time increases with *N*, but decreases with *E*. Figure 3a shows that 〈τ〉 asymptotically follows a scaling law $N^{\alpha }$ with α=1.16(4) in the weak electric field E=0.2. As *E* increases, *α* increases. At the highest field E=32.0, *α* is 1.40(3). We remark that the value of *α* is close to the lower bound 2ν for Rouse dynamics, given by Vocks *et al.* [32], in the weak fields. If the field is strong, it approaches the prediction α=(1+2ν)/(1+ν) [32] because the memory effect becomes important.

The dependence of 〈τ〉 on *E* is shown in Figure 3b. The variation can be characterized by three scaling behaviors in the form: $E^{-\delta }$. *δ* is 1.04(6) in the weak field E<1.0, and increases to be 1.64(5) in the intermediate field region 1.0<E<10.0. It reduces back to 1.0 when E>10.0. The three behaviors can be understood as follows. When the electric field is weak, the chain segments stay in quasi-equilibrium states in both sides of the space at every moment. The system hence responds linearly to the field, with the *δ* exponent equal to 1. As *E* increases, the non-equilibrium effect grows and dominates; the system goes into the so-called trumpet (TP) or stem-flower (SF) regimes [34]. The threading becomes easier than in the previous quasi-equilibrium condition, and the translocation time is shortened, turning to be faster than a linear response (δ>1). The fast decreasing behavior stops when the system goes into the strong stretching regime in the strong electric fields. As a consequence, the scaling behavior returns to the linear track. We remark that the obtained exponent 1.64(5) in the intermediate fields is significantly larger than the predicted 2/(1+ν) for the TP regime and 2ν/(1+ν) for the SF regime [34]. The value is, on the other hand, close to 1/ν predicted by Dubbeldam *et al.* [37] using tensile blob analysis.

**Figure 4:**
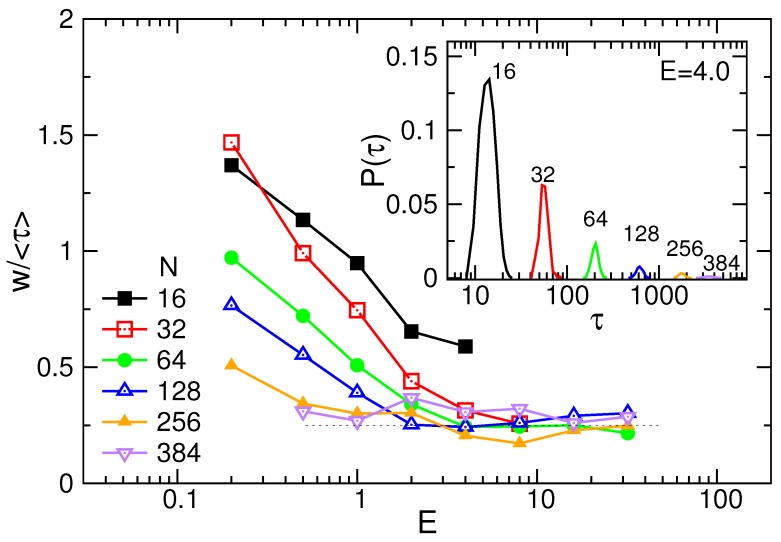
w/〈τ〉 as a function of *E* for different *N*. (Inset) Probability distributions P(τ) of translocation time at E=4.0. The number *N* is indicated near the curve.

We further analyzed the probability distribution P(τ) of translocation time. The results are plotted in the inset of Figure 4, where the field strength *E* was fixed at 4.0, for an example. The distribution displays as a single-peak function, similar to a Gaussian distribution. The peak is lowered and broadened when the chain length increases. Since the horizontal axis is plotted in logarithmic scale for the clarity of the figure, we may have a wrong impression that the width of the distribution does not become wider. At a given chain length *N*, the distribution becomes sharper with increasing *E* (refer to Figure S1 in the Supplementary Materials). To understand the variations, we calculated the ratio of the width *w* of P(τ) to the mean translocation time 〈τ〉. It has been plotted in the main figure as a function of *E* for different *N*. The width *w* is defined as the range of region where the distribution has a value larger than the 1/exp(1)≃0.368 of the peak value. We found that w/〈τ〉 decreases with *E*, and also with *N*. For large *E* and *N*, the ratio tends toward a value of about 0.25.

### 3.2. Chain Size and Positions of Chain Ends

**Figure 5:**
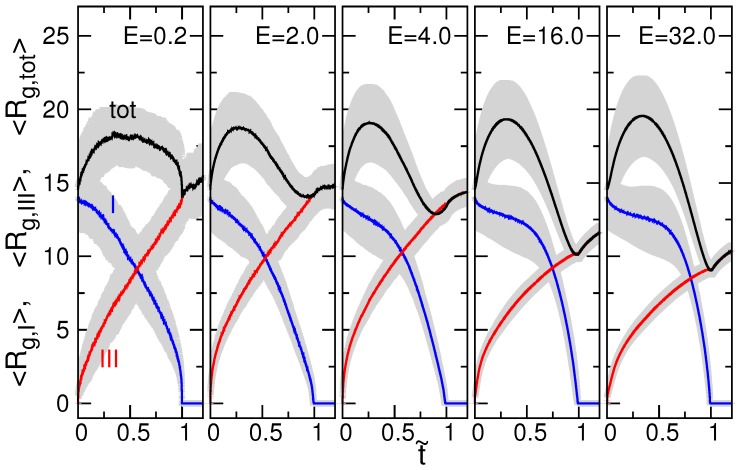
$\tilde{t}$-variation of the averaged $R_{g}$ for the chain of N=128 in the cis-(I), the trans-(III), and the whole (tot) region at E=0.2, 2.0, 4.0, 16.0, and 32.0. The gray-colored region denotes the distribution range of a curve.

To understand the variation of chain size in a translocation process, we calculated the radius of gyration of chain at each instant *t* in the three space regions, defined by $R_{g,R}\left(t\right)={\left(\sum_{i\in R}{\left({\vec{r}}_{i}\left(t\right)-{\vec{r}}_{\mathrm{cm}}\left(t\right)\right)}^{2}/\sum_{i\in R}1\right)}^{1/2}$, where ${\vec{r}}_{i}$ is the position of monomer *i* falling inside the region R and ${\vec{r}}_{\mathrm{cm}}$ is the center of mass of these monomers. Since translocation time is not identical in each process, we normalized the timing *t* by *τ* to be $\tilde{t}=t/\tau$. The radius of gyration was then averaged, over the independent runs, at each normalized $\tilde{t}$-point. The $\tilde{t}$-variation of the averaged $R_{g}$ for N=128 in the cis-region (I), the trans-region (III), and the whole space (tot) are plotted in Figure 5 at different field strengths. The gray-colored region enveloping a curve denotes the distribution range of the curve, which is estimated by the standard deviation.

$\langle R_{g,I}\rangle$ decreases and $\langle R_{g,\mathrm{III}}\rangle$ increases with $\tilde{t}$ simply because the chain threads through the pore, and thus, the chain size decreases in the cis side and increases in the trans side. The total radius of gyration of chain $\langle R_{g,\mathrm{tot}}\rangle$ displays a hump in the middle of the process, resulting from the formation of a dumbbell-like structure of chain separated by the membrane wall. When *E* is weak (refer to the case E=0.2), $\langle R_{g,I}\rangle$ and $\langle R_{g,\mathrm{III}}\rangle$ are approximately mirror-symmetric to each other with respect to the $\tilde{t}=0.5$ point. $\langle R_{g,\mathrm{tot}}\rangle$ is mirror-symmetric to itself too. It shows time-reversible characteristics. The system thus stays in a near-equilibrium state under such a weak perturbation of the driven electric field. As *E* increases, the hump of $\langle R_{g,\mathrm{tot}}\rangle$ is getting higher and occurs at an earlier moment. The $\langle R_{g,I}\rangle$ curve sustains longer near its starting value, while the value of $\langle R_{g,\mathrm{III}}\rangle$ is lowered at $\tilde{t}$ near 1. A fast threading process can produce a compression effect on the chain segment just entering into the trans-region. The faster the threading, the higher the degree of compression, and hence, the smaller the chain size $\langle R_{g,\mathrm{III}}\rangle$. After completion of the threading, the chain size keeps growing. It is a consequence of two effects: (1) relaxation of chain from a compressed state; and (2) diffusion of chain from a near-wall location to the bulk solution.

**Figure 6:**
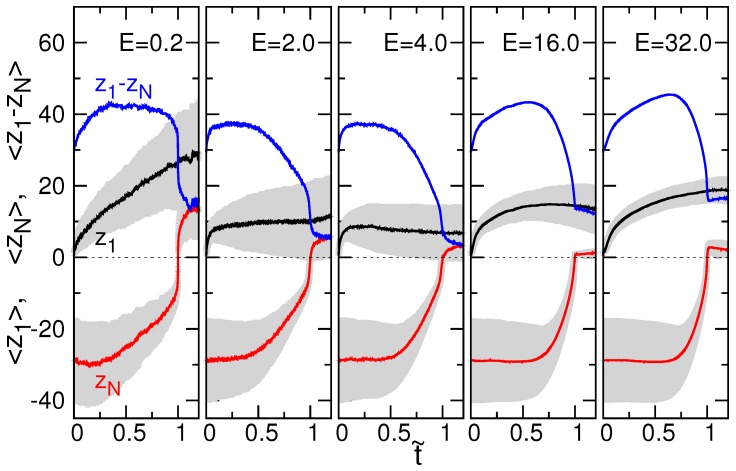
Averaged z-coordinates of chain end, $\langle z_{1}\rangle$ and $\langle z_{N}\rangle$, and the difference $\langle z_{1}-z_{N}\rangle$, as a function of $\tilde{t}$ at different field strengths for N=128. The gray region denotes the distribution range of a curve.

We also studied the position evolution of chain end in a translocation process. Figure 6 shows the averaged z-coordinates of chain end, $\langle z_{1}\rangle$ and $\langle z_{N}\rangle$, as a function of $\tilde{t}$.

The heading end $\langle z_{1}\rangle$ increases continuously for E=0.2, because the slow threading rate gives enough time for the chain to relax and to diffuse away from the wall. As *E* increases (up to 4.0), the degree of increasing diminishes since the chain segment has not yet been relaxed against the resistance (pressure) of the monomers and ions presented in the trans-region. Starting from E=4.0, a hump appears on the $\langle z_{1}\rangle$ curve and grows with field strength. It is because of the inertia effect, in which the monomers are more accelerated in the channel by a stronger field and hence “projected” farther away from the pore, when exiting the channel. Owing to the chain connectivity, a second effect comes in, which swings the chain end tangentially toward the longitudinal direction and results in a position close to the wall with the evolution of time. The value of $\langle z_{1}\rangle$ thus decreases.

On the other hand, the tail monomer is not influenced instantly by the threading. The increase of $\langle z_{N}\rangle$ becomes obvious only when the contour distance of the tail monomer to the pore entrance decreases to be comparable to the direct distance. This effect is more important when the electric field is strong, for the cases E=16.0 and 32.0, where $\langle z_{N}\rangle$ maintains approximately at the same position until $\tilde{t}≃0.75$. The averaged difference $\langle z_{1}-z_{N}\rangle$ has been plotted in the figure. A sudden drop was observed near $\tilde{t}=1$, suggesting a surge of tension force near the end of a threading process.

The variations of chain size and *z*-coordinates of chain end for longer chains, N=256 and N=384, have been given in the Supplementary Materials for comparison.

### 3.3. Translocation Coordinate

**Figure 7:**
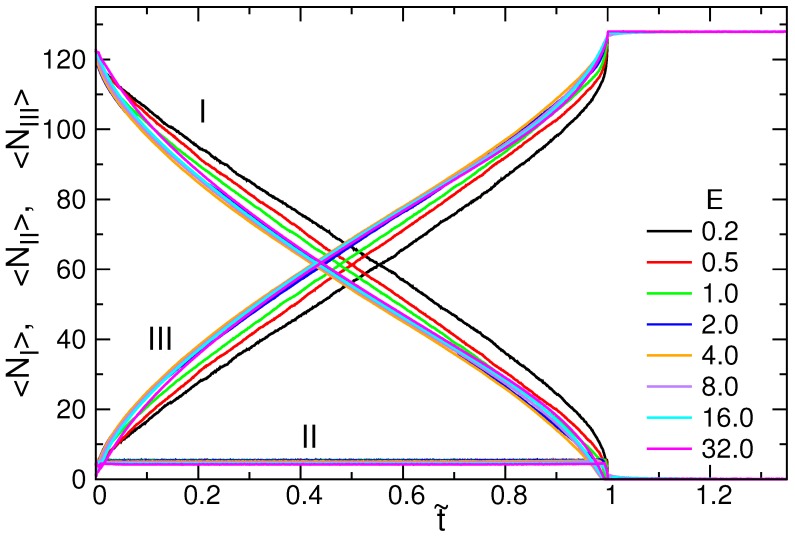
$\tilde{t}$-variation of the averaged $N_{m}$ for N=128 in the cis-region (I); the pore-region (II); and the trans-region (III). The field strength *E* is indicated in the figure.

The variations of the number of monomer $N_{m}$ in the cis-region (I), the pore-region (II), and the trans-region (III) were investigated. $N_{m,\mathrm{III}}$ is also called “the translocation coordinate”, usually denoted by *s* in literature [16,31]. The value describes the progress of translocation, and evolves gradually from 1 to *N* (refer to Figure S5 in Supplementary Materials). The zigzagged $N_{m,\mathrm{III}}$ curves reveal the diffusion characteristics of translocation. To extract the drifting part of the dynamics, we calculated the average of the curves. The results are shown in Figure 7 for N=128.

$\langle N_{m,\mathrm{III}}\rangle$ monotonically increases for $\tilde{t}\leq 1$. When *E* increases, the entire curve first moves upwards, up to E=4.0, and then moves downwards for the higher *E*. Therefore, the translocation progresses relatively more in advance in E=4.0 at every $\tilde{t}$-moment. The average number of monomers in the pore region, $\langle N_{m,\mathrm{II}}\rangle$, is about four (also plotted in the figure). Consequently, $\langle N_{m,I}\rangle$, in the cis-region, monotonically decreases during the threading process, because $\langle N_{m,I}\rangle +\langle N_{m,\mathrm{II}}\rangle +\langle N_{m,\mathrm{III}}\rangle =N$.

**Figure 8:**
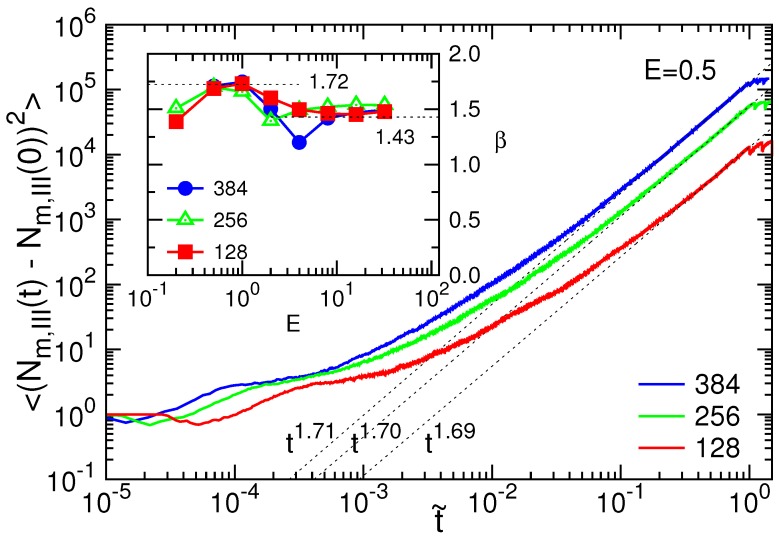
MSD $\langle {\left(N_{m,\mathrm{III}}\left(t\right)-N_{m,\mathrm{III}}\left(0\right)\right)}^{2}\rangle$ at E=0.5, plotted with the normalized time $\tilde{t}$, for N=128, 256, and 384. (Inset) Exponent *β* vs. *E* for different *N*.

We further investigated the mean square displacement (MSD) of the translocation coordinate, $\langle {\left(N_{m,\mathrm{III}}\left(t\right)-N_{m,\mathrm{III}}\left(0\right)\right)}^{2}\rangle$. At t=τ, $N_{m,\mathrm{III}}$ is equal to *N*. It implies $\langle {\left(N_{m,\mathrm{III}}\left(\tau \right)-N_{m,\mathrm{III}}\left(0\right)\right)}^{2}\rangle ∼N^{2}∼{\langle \tau \rangle }^{2/\alpha }$. Therefore, the MSD is anticipated to show some scaling behavior near t=〈τ〉 under the form $t^{\beta }$ with the exponent β=2/α. Our simulations did show this scaling behavior. An example is given in Figure 8 for the long chain cases (N=128, 256, and 384) driven by E=0.5, where the MSD is plotted with the normalized time $\tilde{t}$.

By fitting the scaling in the range $0.5\leq \tilde{t}\leq 1.0$, we calculated the exponent *β*. The results are shown in the inset of the figure. We found that aside from the fluctuation, the value of *β* is close to 1.75 in the weak fields and decreases to a value around 1.5 in the strong fields. It is in agreement with Figure 3a, where the exponent α=1.16 for the weak field yields β=2/α=1.72, and α=1.40 for the strong field yields β=1.43. The MSD calculation double-checks these results.

### 3.4. Ion Condensation

**Figure 9:**
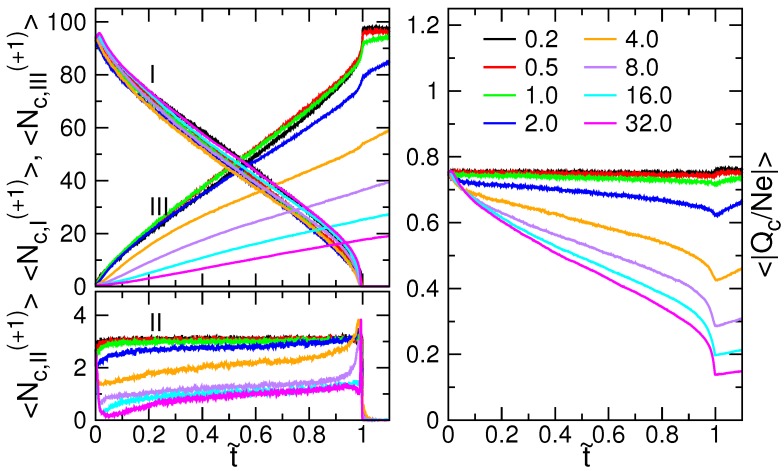
(Left) Variations of the average number of condensed counterions $N_{c}^{\left(+1\right)}$ in the cis-region (I), the pore-region (II), and the trans-region (III) for N=128. (Right) Fraction of charges neutralized on the chain, $\langle |Q_{c}/Ne|\rangle$, during a translocation process. The field strength *E* is indicated in the legend.

In polyelectrolyte solutions, a considerable number of dissociated ions condense onto chains owing to electrostatic interaction. According to Manning theory [90,91,102], the condensed ions effectively reduces the absolute value of the line charge density of the chain to $|\Gamma^{*}|=e/\lambda_{B}$. To study this topic, we chose N=128 as an example and monitored the number of ions condensing on the chain during a translocation process. A distance criterion was used to determine the ion condensation: an ion is said to be condensed on a chain if the distance of the ion to the nearest monomer on the chain is smaller than the Bjerrum length $\lambda_{B}=3$ [103,104,105,106]. We counted the numbers of the condensed counter-ions ($N_{c}^{\left(+1\right)}$) and co-ions ($N_{c}^{\left(-1\right)}$) in the three space regions, cis (I), pore (II), and trans (III), separately. Since the chain is negatively charged, the majority of the condensed ions are the counterions ((+1)-ions). In the left panel of Figure 9, we plot the average numbers of the counterions, $\langle N_{c,I}^{\left(+1\right)}\rangle$, $\langle N_{c,\mathrm{II}}^{\left(+1\right)}\rangle$, and $\langle N_{c,\mathrm{III}}^{\left(+1\right)}\rangle$, as function of $\tilde{t}$.

In the course of translocation, $\langle N_{c,I}^{\left(+1\right)}\rangle$ decreases and $\langle N_{c,\mathrm{III}}^{\left(+1\right)}\rangle$ increases, which follows, respectively, the decreasing trend of $\langle N_{m,I}\rangle$ and the increasing trend of $\langle N_{m,\mathrm{III}}\rangle$. However, increasing the field strength moves the $\langle N_{c,\mathrm{III}}^{\left(+1\right)}\rangle$-curve downward significantly when E≥2.0, while the $\langle N_{c,I}^{\left(+1\right)}\rangle$-curve is largely insensitive to the electric field. It is because the counterions have not enough time to condense thoroughly onto the chain segment in the trans side when the translocation time is shortened. The stronger the field, the smaller the number of $\langle N_{c,\mathrm{III}}^{\left(+1\right)}\rangle$. In the pore region, $\langle N_{c,\mathrm{II}}^{\left(+1\right)}\rangle$ is around 3 for small *E* (refer to the left-bottom panel of the figure). If *E* is strong such as E=32.0, the value is reduced to about 1.

We further calculated the total charges of the condensed ions on the chain, $Q_{c}=eN_{c}^{\left(+1\right)}+\left(-e\right)N_{c}^{\left(-1\right)}$. The absolute ratio of the average total charges to the chain bare charges, $\langle |Q_{c}/Ne|\rangle$, gives the information about the fraction of chain charge neutralized by the condensed ions, and has been plotted in the right panel of Figure 9.

We can see that $\langle |Q_{c}/Ne|\rangle$ acquires a value of 0.77 at the starting point $\tilde{t}=0$. It decreases very gently during the threading process when E<1.0, and regains immediately the starting value once the process has been completed. The contour length of chain in this case is ℓ=127.9σ. The effective line charge density is thus calculated, and yields $|(-Ne+Q_{c})/\ell |=0.23e/\sigma$. This value is about 30% smaller than the value of Manning’s theory, which predicts $|\Gamma^{*}|≃0.33e/\sigma$. Therefore, the degree of ion condensation is higher in the simulations. The discrepancy can be attributed to the usages of (1) the flexible chain; (2) the finite salt concentration; and (3) the wall, in the simulations. These settings are outside the hypothesis of Manning’s theory, which is designated for a rigid, infinitely-long polyelectrolyte in a dilute bulk solution without added salt [102].

The nearly-constant $Q_{c}$ in the weak fields shows that the total number of condensed ions on the chain is roughly constant. If the field strength is strong (e.g., E≥2.0), the condensed ions can be peeled off the chain before entering the pore. It results in a reduction of $\langle |Q_{c}/Ne|\rangle$. At a fixed *E*, the $\langle |Q_{c}/Ne|\rangle$ curve shows a higher decreasing rate near the end of a process ($\tilde{t}=1$). The peeling effect is gone once the process is completed. $Q_{c}$ re-increases because of the re-condensation of the counterions in the trans-region.

The results of ion condensation for longer chain N=256 and N=384 have been given in Figure S4 of the Supplementary Materials for reference.

### 3.5. Probability Distributions and Density Distributions

**Figure 10:**
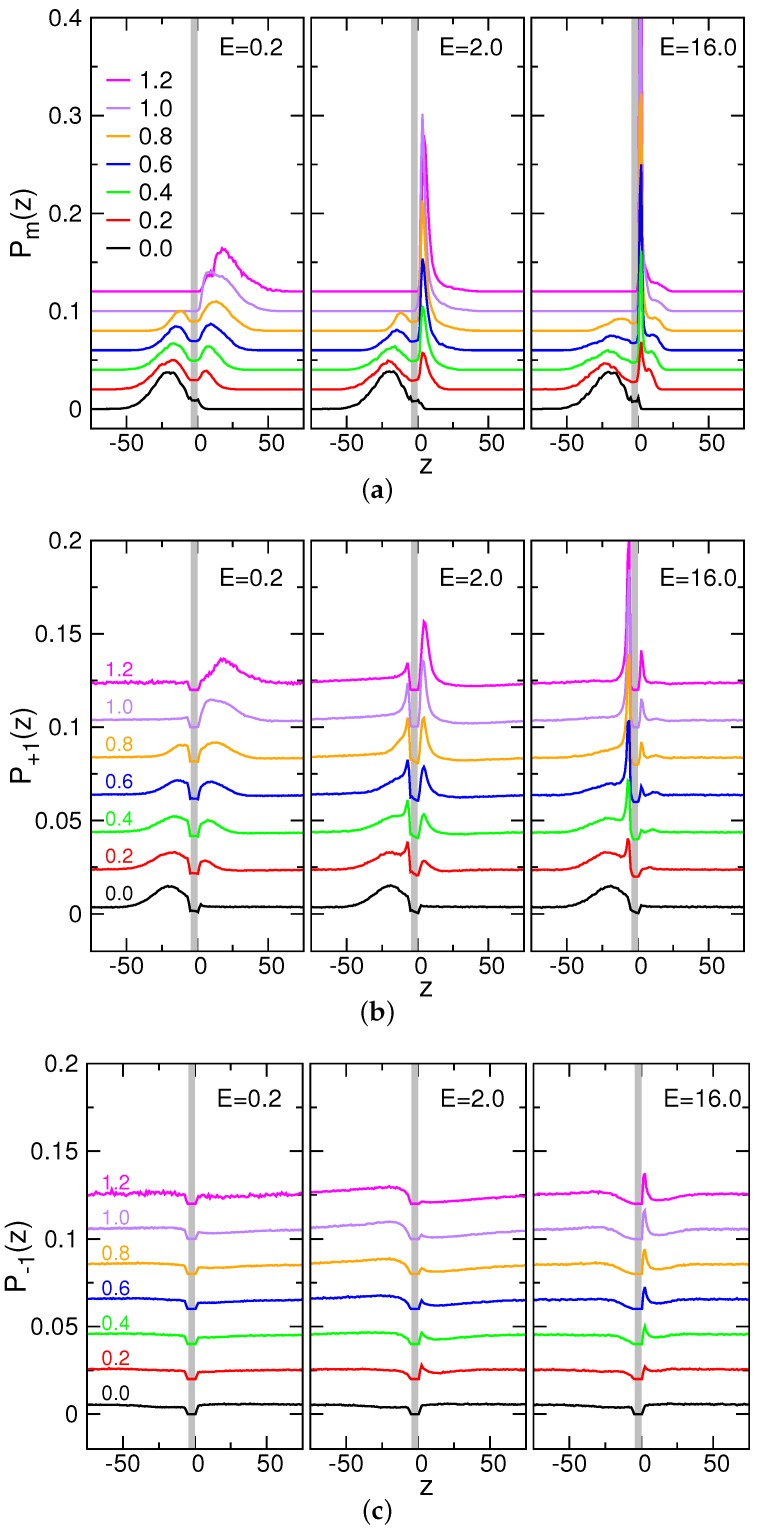
Probability distributions in the *z*-direction for (**a**) monomers; (**b**) counterions; and (**c**) coions; at E=0.2, 2.0, and 16.0 (N=128). The gray color denotes the *z*-location of the pore. The value given near a curve is the normalized time $\tilde{t}$ at which the probability density was calculated. For clarity, the curves have been shifted upward with a fixed step value, one curve after the others.

In order to understand the evolution of different components in the system in translocation, we calculated the probability density distributions in the *z*-direction. Figure 10 shows the distribution functions for monomers ($P_{m}\left(z\right)$), counterions ($P_{+1}\left(z\right)$), and coions ($P_{-1}\left(z\right)$), at three different field strengths, for the chain length N=128. Each distribution was calculated within a time interval $\left[\tilde{t},\tilde{t}+0.01\right]$ with the value of $\tilde{t}$ given near the curve. We have shifted the curves upward with a fixed step value, one curve after the others, for the clarity of the plot.

At $\tilde{t}=0$, $P_{m}\left(z\right)$ displays a peak in the cis side (left to the gray-colored (pore) region in the figure). The peak-to-wall distance was calculated and has a value of 16.4. Since the distance is comparable to the size of chain $\langle R_{g,I}\rangle =14.0\pm 1.9$, the starting configuration is similar to a coiled chain tethered on a surface.

First, pay attention to the weak field case E=0.2. We can see that a second peak is formed gradually in the trans side (right to the gray region in the figure), and slowly propagates to the right, away from the wall, as time evolves. The counterion distribution $P_{+1}\left(z\right)$ follows basically the profile of $P_{m}\left(z\right)$ because of ion condensation, whereas the coion distribution $P_{-1}\left(z\right)$ remains flat in the cis- and trans-regions. In the stronger field E=2.0, the second peak of $P_{m}\left(z\right)$ is formed close to the wall and is much sharper than in the weak field. The threading rate is so high that the chain segment entering the trans side has not yet been relaxed. Therefore, monomers gathered near the exit of the pore. For the very strong field E=16.0, a small hump appears next to the second peak. It results from the diffusive motion of monomers in the radial direction, departing from the pore exit. The very sharp second peak shows that the monomers are in contact with the wall and diffuse also in the lateral direction. We noticed that the first peak in the cis side is flattened in comparison with the one for the case E=0.2. It is because the fast threading speed causes a tight tension and hence elongates the chain. The counterion distribution $P_{+1}\left(z\right)$ for the cases E=2.0 and E=16.0 exhibits a peak in the cis-region in contact with the wall. It is related to the peeling of the condensed counterions when the chain enters to the pore. The peak formed on the right-hand side of the wall results from the re-condensation of counterions in the trans-region, attracted by the monomers. The peak height is lower for the case E=16.0 than for E=2.0 because the real timing $t=\tilde{t}\langle \tau \rangle$ is much shorter for E=16.0, and thus, less counterions were able to re-condense in the shorter time duration. For coions, there appears a surge of $P_{+1}\left(z\right)$ in the cis side near the wall, for E=2.0 and E=16.0. The fast withdrawing of chain liberates the space for the coions to come in, which forms the surge, particularly when $\tilde{t}$ is near 1. A small peak displayed in the trans side shows a trap of the coions, which results from a combined interaction with condensed counterions and monomers.

**Figure 11:**
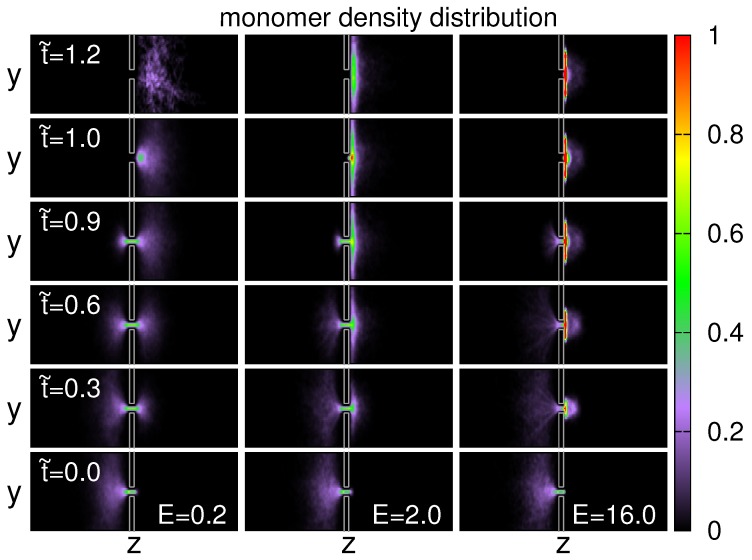
Density distributions of monomer, viewed along *x*-axis, at (Left) E=0.2, (Middle) E=2.0, and (Right) E=16.0. The chain length is N=128. The value of $\tilde{t}$ is indicated in the figure.

Moreover, we investigated the density distribution of monomers to understand the details of monomer transport across the pore. Figure 11 shows the results at the three electric fields, viewed from the *x*-direction, also for the case N=128.

At $\tilde{t}=0$ and E=0.2, the density distribution exhibits a profile similar to a shuttlecock, with the body part lying in the cis-region. As time goes by, a second “shuttlecock” appears in the trans-region and the body size grows with time. It is worth noticing that the density profiles at $\tilde{t}$ and at $1-\tilde{t}$ look mirror-symmetric to each other, suggesting a near-reversible process. For the case of stronger field E=2.0, the mirror-symmetry is broken. The density profile in the trans side displays a pancake-like silhouette, in contact with the separation wall. It is obviously a process far from equilibrium. If the electric field is very strong (E=16.0), the fast threading rate leads the appearance of several “stem lines” in the cis-region towards the head of the shuttlecock. These lines are a consequence of the elongated chain due to tight tension. In the trans side, the density profile is a flat pancake with a hump cloud over it. It shows two dominated motions: (1) relaxation of chain monomers in the lateral direction; (2) diffusion of monomers radially from the pore.

## 4. Conclusions

Using Langevin dynamics simulations, we have investigated charged polymers driven through a nanopore by electric fields. The mean translocation time was found to follow a scaling law $\langle \tau \rangle ∼N^{\alpha }E^{-\delta }$ in the long-chain limit, with *α* and *δ* depending on *E*. The exponent *α* is equal to 1.16(4) in a weak field, increases with *E*, and acquires a value 1.40(3) when the field is strong. The two limiting values are in agreement, respectively, with the lower bound exponent 2ν and the exponent (1+2ν)/(1+ν) (when the memory effect is important), for Rouse dynamics [32]. *δ* is 1 in the weak and in the strong fields. For the field strength in between, *δ* is 1.64(5), which is close to the prediction 1/ν [37]. We have calculated the mean square displacement of the translocation coordinate and found that it scales as $t^{\beta }$ when *t* is close to *τ*. The extracted *β* is in accordance with the relation αβ=2, which doubly verified the dependence of *α* on *E*. The study of probability distribution P(τ) revealed that the distribution width increases with *N*. However, in the strong-field limit, the width rests at a value of about 25% of the mean translocation time. In the weak fields, the threading of chain can be regarded as a near-equilibrium process because the radii of gyration for different chain segments displayed certain time-reversible symmetries. These symmetries were broken when the field strength got strong and therefore, the system entered to the domain of far-from-equilibrium. How the positions of chain ends evolved in a translocation process have been investigated systematically in different field strengths.

Since the ions were modeled explicitly, we were able to study ion condensation on a threading chain. The total charges of condensed ions maintained roughly at a constant value in the weak fields. If the field became strong, the counterions were stripped off the chain when the chain passed the pore, leading to a decrease of the charges during a process. We have studied the probability distributions for monomer, counterion, and coion. The evolution of these distributions showed how the different components of system varied with time in the longitudinal direction. In order to obtain the transverse information, we have calculated the density distributions. The results revealed that the monomers entered into the trans-region were gathered firstly near the wall, due to fast threading rate by a strong field. The accumulated density were then relaxed and formed a pancake profile with a hump cloud over it. The information and pictures obtained here provide a deep insight of single polyelectrolytes threading through a nanopore, forced by electric fields. The concept might be used to develop or understand advanced materials, for example, with polymers threading into nanoporous materials [107,108].

## Figures and Tables

**Table 1:** Parameters of simulation, in the (*σ*, *m*, $\tau_{u}$, *e*) unit.

Parameter	Value	Description
($\sigma_{\text{bb}}$, $\varepsilon_{\text{bb}}$)	(1.0, 1.2)	Excluded volume interaction
($\sigma_{\text{bw}}$, $\varepsilon_{\text{bw}}$)	(1.5, 2.5)	Excluded volume interaction
(*k*, $r_{0}$)	(600.0, 1.0)	Bond connectivity
$\lambda_{B}$	3.0	Bjerrum length (for the Coulomb interaction)
(*ζ*, *T*)	(1.0, 1.0)	Settings for the Langevin equation
*N*	16–384	Number of monomers on a chain
*E*	0.2–32.0	Strength of the applied electric field
—	2.25	Radius of pore
—	4.5	Length of pore
—	48.0×49.36×200.0	Dimension of box

## Supplementary Materials

The following are available online at www.mdpi.com/2073-4360/8/3/73/s1, Figure S1: probability distribution P(τ) at different *E*, Figure S2: variation of $\langle R_{g}\rangle$ at different *N*, Figure S3: variations of $\langle z_{1}\rangle$ and $\langle z_{N}\rangle$ at different *N*, Figure S4: variations of $\langle N_{c}^{\left(+1\right)}\rangle$ and $\langle |Q_{c}/Ne|\rangle$ at different *N*, Figure S5: variations of translocation coordinate (raw data).

## List of Symbols:

〈X〉	Mean value of physical quantity *X*
$X_{I}$, $X_{\mathrm{II}}$, $X_{\mathrm{III}}$	Values of physical quantity *X* in the cis-region (I), pore-region (II), trans-region(III)
*α*	Exponent of the scaling behavior $\langle \tau \rangle ∼N^{\alpha }$
*β*	Exponent of the scaling behavior $\langle {\left(N_{m,\mathrm{III}}\left(t\right)-N_{m,\mathrm{III}}\left(0\right)\right)}^{2}\rangle ∼t^{\beta }$
*δ*	Exponent of the scaling behavior $\langle \tau \rangle ∼E^{-\delta }$
*E*	Strength of electric field (applied only in the pore region)
*e*	Elementary charge
*ϵ*	Dielectric constant of solvent
$\varepsilon_{\text{bb}}$, $\varepsilon_{\text{bw}}$	Energy parameters of Lennard-Jones potential for bead-bead (bb) and bead-wall (bw) interactions
${\vec{\eta }}_{i}$	Stochastic force acting on the bead *i*
$\left|\Gamma^{*}\right|$	$e/\lambda_{B}$
*k*	Spring constant of bond
$k_{B}$	Boltzmann constant
*ℓ*	Contour length of chain
$\lambda_{B}$	Bjerrum length
*m*	Mass unit of simulation
*N*	Chain length
$N_{c}^{\left(+1\right)}$, $N_{c}^{\left(-1\right)}$	Numbers of condensed (+1)-ions, condensed (−1)-ions
$N_{m}$	Number of monomers
*ν*	Flory exponent
P(τ)	Probability distribution of translocation time
$P_{m}\left(z\right)$, $P_{+1}\left(z\right)$, $P_{-1}\left(z\right)$	Probability density distributions in *z*-direction for monomer, (+1)-ions, (−1)-ions
$Q_{c}$	Total charges of condensed ions
$R_{g}$	Radius of gyration
*r*	Distance between two beads
$r_{0}$	Equilibrium length of bond
${\vec{r}}_{i}$	Position vector of the bead *i*
*σ*	Length unit of simulation
$\sigma_{\text{bb}}$, $\sigma_{\text{bw}}$	Length parameters of Lennard-Jones potential for bead-bead (bb) and bead-wall (bw) interactions
*T*	Temperature
*t*	Time
$\tilde{t}$	Normalized time $\left(\tilde{t}=t/\tau \right)$
*τ*	Translocation time
$\tau_{u}$	Time unit of simulation
*w*	Width of P(τ)
$Z_{i}$	Valence of the bead *i*
$z_{1}$, $z_{N}$	*z*-coordinates of chain head-end and tail-end
*ζ*	Friction coefficient
