# Supplementary material for: Polyelectrolyte Threading through a Nanopore
Source: Polymers (Basel). 2016 Mar 3;8(3):73. doi: 10.3390/polym8030073 (PMC6432567; doi:10.3390/polym8030073)
Supplement: Supplementary file 1 [file polymers-08-00073-s001.pdf]

Supplementary Materials: Polyelectrolyte Threading through a Nanopore

Pai-Yi Hsiao

The probability distribution of translocation time $P(\tau)$ is a single-peak function (Figure S1). The peak is lowered and broadened as the chain length N increases. At a given N, the distribution becomes sharper with increasing E.

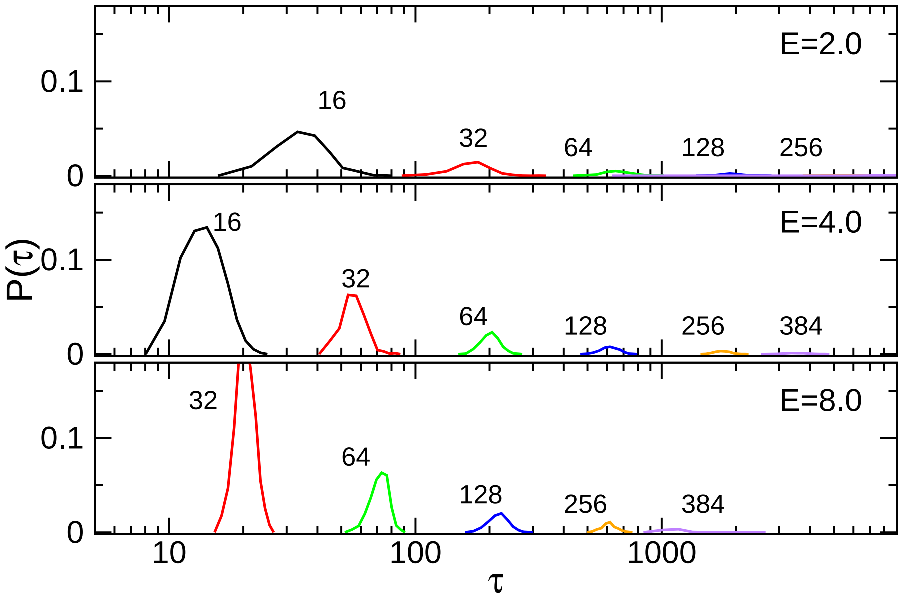

Figure S1. Probability distribution $P(\tau)$ of translocation time at $E = 2.0$, 4.0, and 8.0. The chain length N is indicated near the curves.

How the chain size $\langle R_g \rangle$, the z-coordinates of chain end $\langle z_1 \rangle$, $\langle z_N \rangle$, the number of condensed counterions $\langle N_c^{(+1)} \rangle$, and the fraction of charge neutralization $\langle |Q_c/Ne| \rangle$ depend on the chain length N can be seen through a comparison of the cases (a) $N = 128$, (b) $N = 256$, (c) $N = 384$ in Figures S2–S4. The results show similar trend of variation during a translocation process, for different chain lengths.

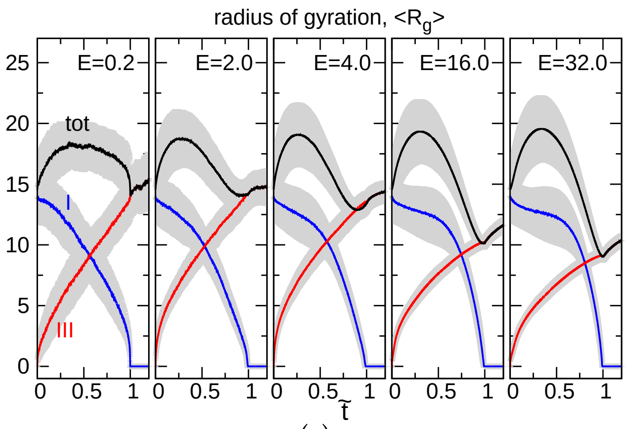

(**a**)

Figure S2. *Cont.*

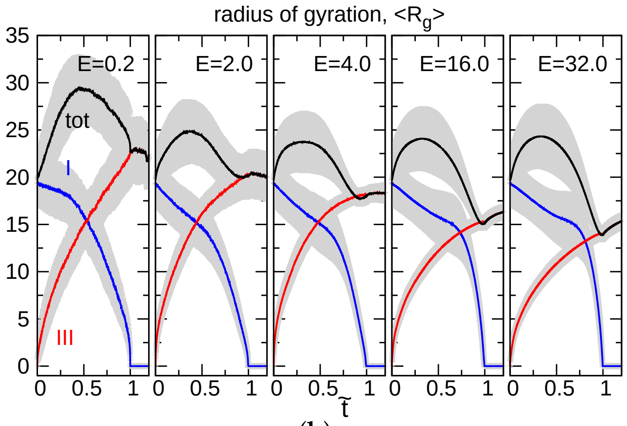

(**b**)

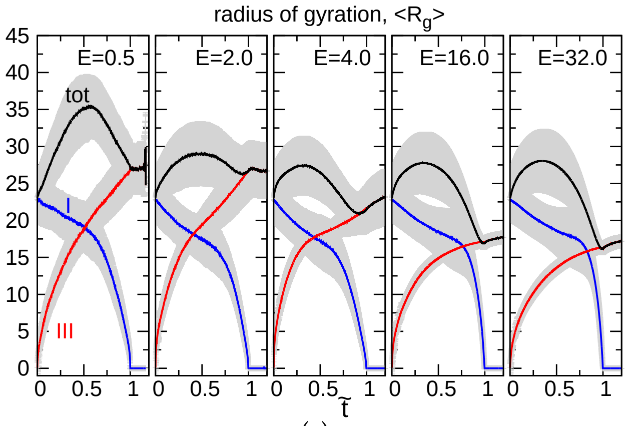

(**c**)

Figure S2. Variation of $\langle R_g \rangle$ in the cis- (I), the trans- (III), and the whole (tot) region at different E fields, for (**a**) $N = 128$; (**b**) $N = 256$; and (**c**) $N = 384$. The gray-colored region denotes the error of a curve.

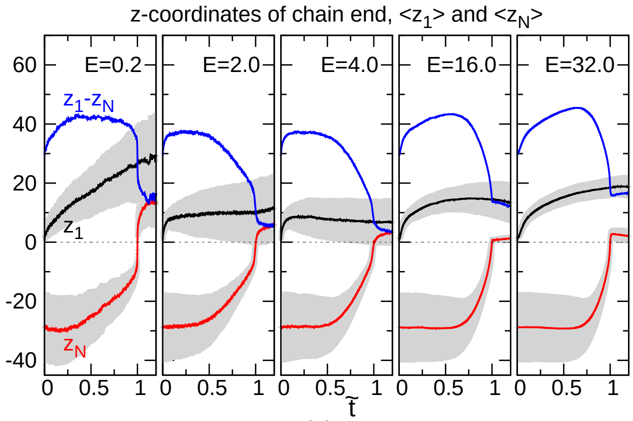

(**a**)

Figure S3. *Cont.*

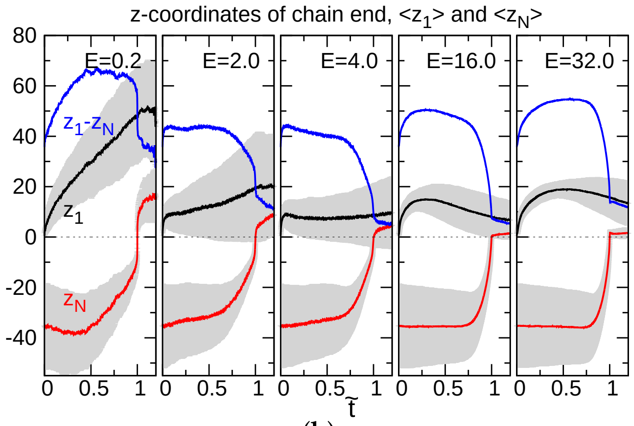

(**b**)

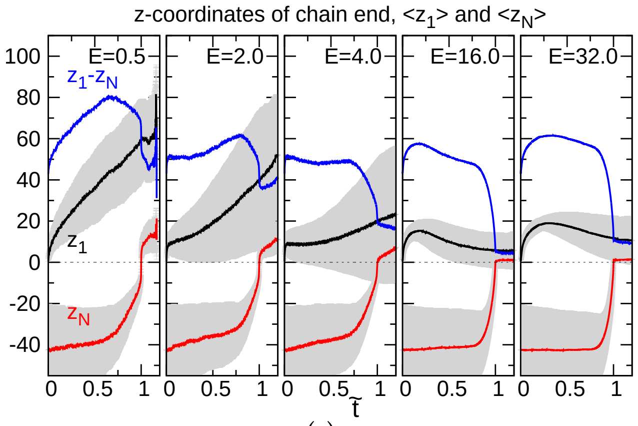

(**c**)

Figure S3. Averaged z-coordinates of chain end, $\langle z_1 \rangle$ and $\langle z_N \rangle$, and the difference $\langle z_1 - z_N \rangle$, as a function of $\tilde{t}$ at different field strength for (**a**) $N = 128$; (**b**) $N = 256$; and (**c**) $N = 384$.

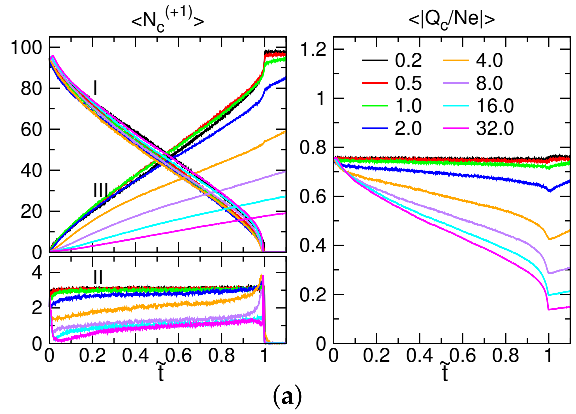

(**a**)

Figure S4. *Cont.*

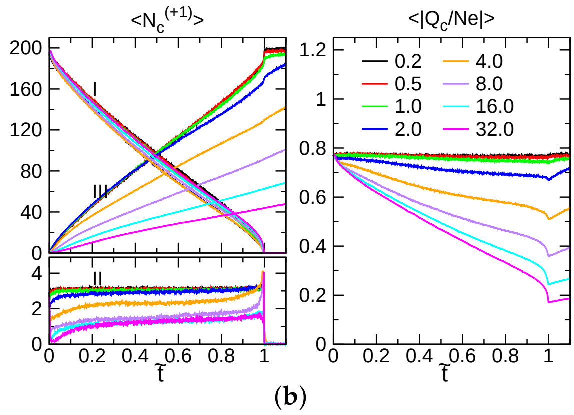

(**b**)

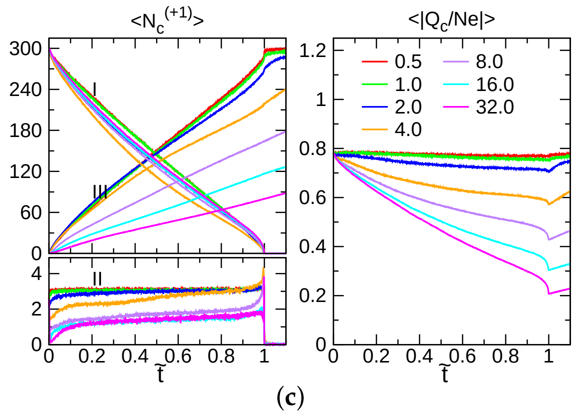

(**c**)

Figure S4. (Left) Variations of mean number of condensed counterions $N_c^{(+1)}$ in the cis-region (I), the pore-region (II), and the trans-region (III) for (**a**) $N = 128$; (**b**) $N = 256$; and (**c**) $N = 384$. (Right) Fraction of charges neutralized on the chain, $\langle |Q_c/Ne| \rangle$, during a translocation process for (**a**) $N = 128$; (**b**) $N = 256$; and (**c**) $N = 384$. The field strength E is indicated in the legend.

The zigzagged curves of the translocation coordinate $N_{m,III}$ show diffusion characteristics of translocation when the chain threads through the pore (in Figure S5). In contrast, the variation of the averaged translocation coordinate $\langle N_{m,III} \rangle$ is a smooth, monotonically increased function. It depicts the drifting behavior of the chain forced by the applied electric field.

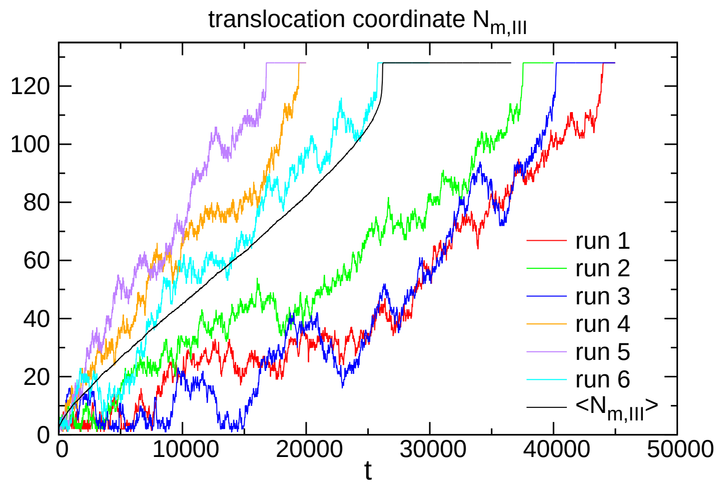

Figure S5. Variation of translocation coordinate for $N = 128$ at $E = 0.2$: raw data (run 1 – run 6) *vs.* the averaged behavior $\langle N_{m,III} \rangle$
